# Multi-Channel High-Performance Absorber Based on SiC-Photonic Crystal Heterostructure-SiC Structure

**DOI:** 10.3390/nano12020289

**Published:** 2022-01-17

**Authors:** Jing Han, Jijuan Jiang, Tong Wu, Yang Gao, Yachen Gao

**Affiliations:** 1 Electronic Engineering College, Heilongjiang University, Harbin 150080, China; hanjing1980@163.com (J.H.); 02781@qqhru.edu.cn (J.J.); 124wutong@163.com (T.W.); 2 College of Science, Heilongjiang Institute of Technology, Harbin 150050, China; 3 College of Communication and Electronic Engineering, Qiqihar University, Qiqihar 161000, China

**Keywords:** optical Tamm state, Tamm phonon polariton, mode coupling, epsilon-near-zero phonon polariton, multi-channel absorber

## Abstract

The multi-channel high-efficiency absorber in the mid-infrared band has broad application prospects. Here, we propose an SiC-photonic crystal (PhC) heterostructure-SiC structure to realize the absorber. The absorption characteristics of the structure are studied theoretically. The results show that the structure can achieve high-efficiency multi-channel absorption in the mid-infrared range. The absorption peaks come from the coupling of the dual Tamm phonon polariton (TPhP) mode formed at the interface between the two SiC layers and the photonic crystal, and the optical Tamm state (OTS) mode formed in the PhC heterostructure. By adjusting the thickness of the air dielectric layer and the period of the PhC in the heterostructure, the mode coupling intensity can be regulated; thereby, the position and intensity of the absorption peak can be adjusted. In addition, the absorption peaks of TE and TM polarized light can be controlled by changing the incident angle. Adjusting the incident angle can also control the excitation and intensity of the epsilon-near-zero (ENZ) phonon polariton mode produced by TM polarized light. This kind of light absorber may have potential applications in sensors, filters, modulators, switches, thermal radiators, and so on.

## 1. Introduction

Efficient infrared absorption has a wide range of applications, including sensors [1], photodetectors [2], thermal emitters [3], infrared imaging [4], etc. Compared with single-channel absorbers, multi-channel optical absorbers have a broader application field, so people have always hoped to achieve multi-channel controllable and high-efficiency absorption in a wide wavelength range. Currently, the multi-channel optical absorbers are mainly based on electromagnetic resonance [5,6], surface plasmon resonance [7,8], and optical Tamm state (OTS) [9]. OTS is a localized interface mode. One-dimensional photonic crystal (PhC) heterostructure and metal-PhC structure are two commonly used structures to produce OTS [10]. OTS produced near the metal-PhC interface is also called Tamm plasmon polariton (TPP) [11]. OTS or TPP can be excited directly by TE or TM polarized light, even under normal incident condition. Changing the dielectric layer in the PhC heterostructure can adjust the position and number of OTS resonance peaks and the interaction with other modes, enabling adjustment of the transmission and absorption characteristics of the structure [12]. Many schemes for building OTS mode absorbers have been proposed [13,14].

In the mid-infrared band, the use of polar dielectrics instead of metals has been explored. Similar to metals, polar dielectrics show high reflectivity and a negative real part of the dielectric constant in the reststrahlen band. The electromagnetic wave incident on the surface of polar dielectrics may excite evanescent waves in the reststrahlen band, producing surface phonon polariton (SPhP) [15,16]. If a polar dielectric is used to replace the metal of the metal-PhC structure, Tamm phonon polariton (TPhP) will be produced similar to TPP [17].

In the interaction between light and matter, the Fano resonance and the effect similar to Rabi splitting can be easily observed [18,19,20,21]. Fano resonance is usually caused by interference between high-quality factor (high-Q) mode and low-quality factor (low-Q) mode. It usually forms a narrow non-absorption band in the single-band absorption spectrum. The Rabi splitting effect is a strong coupling between two similar Q resonant modes [22]. The strong coupling provides a new method for multi-band resonance. It has been widely studied to achieve multi-band absorption by coupling OTS with other modes [21]. However, there are few studies on the multi-channel high-efficiency absorption formed by the coupling of OTS and TPhP.

In this paper, an SiC-PhC heterostructure-SiC structure is designed, and a multi-channel high-efficiency absorption in the mid-infrared band is realized by the exciting and coupling of multiple modes. The finite-difference time-domain (FDTD) method is used to simulate the transmission/absorption spectrum and electric field distribution of the structure. The transfer matrix method (TMM) and coupled harmonic oscillator model are used for theoretical analysis. The results show that the number, position, intensity, and coupling of the absorption peaks can be controlled by adjusting the period of the PhC, the thickness of the air layer in the heterostructure, the incident angle of the incident light, and the polarization state of the incident light. The proposed multi-channel absorber has potential applications in sensors, filters, and thermal radiators.

## 2. Structure and Theory

**Figure 1:**
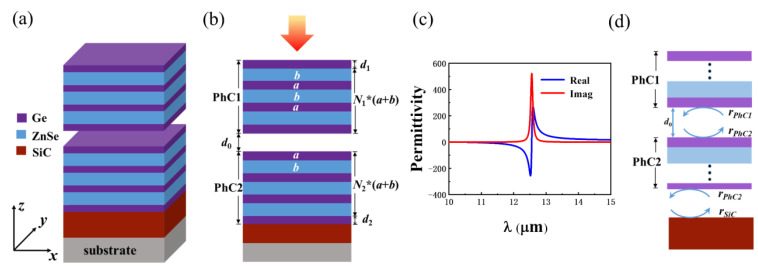
(**a**) Schematic diagram of the structure of PhC1-air layer-PhC2-SiC. *N*_1_*(*a + b*) and *N*_2_*(*a + b*) indicate that the number of periodic layers of PhC is *N*_1_ and *N*_2_, respectively. (**b**) Side view of the structure. (**c**) The dependence of the real part and the imaginary part of the permittivity of SiC on the wavelength. (**d**) The 2D view of the structure used to generate OTS and TPhP.

Figure 1a,b shows a part of the proposed structure, in which PhC2 is deposited on the SiC film layer and the substrate. PhC1 is used as a suspended film, and the distance between PhC1 and PhC2 can be tuned finely by a nanopositioning system. The structure extends infinitely in the x and y directions. The heterostructure consists of PhC1 and PhC2, which are alternately composed of Ge and ZnSe, and the refractive index is 4.0 and 2.4, respectively [23,24]. The thicknesses of the composite layer are a = 0.6 μm and b = 1.05 μm, respectively. d_1_ = 0.6 μm and d_2_ = 0.4 μm represent the underlayer thicknesses of the PhC1 and PhC2, and d_0_ is the thickness of the air layer at the interface of the heterostructure. N_1_ and N_2_ represent the numbers of periods of PhC1 and PhC2. TE or TM polarized light is incident, with an angle of $\theta =0^{\circ }$.

SiC is a polar dielectric material, and its dielectric constant can be described by the Lorentz oscillator model [25]:

(1) $$\varepsilon (\omega )=\varepsilon_{\infty }(1+\frac{\omega_{LO}^{2}-\omega_{TO}^{2}}{\omega_{TO}^{2}-\omega^{2}-i\gamma \omega }),$$

where $\varepsilon_{\infty }$ is the high-frequency dielectric constant, ω is the angular frequency of the incident light, $\omega_{LO}$ and $\omega_{TO}$ are the longitudinal optical phonon frequency and the transverse optical phonon frequency, respectively, and γ is the phonon damping coefficient. For SiC, $\varepsilon_{\infty }=6.56$, $\omega_{LO}=973{\mathrm{cm}}^{-1}$, $\omega_{TO}=797{\mathrm{cm}}^{-1}$, and $\gamma =4.76{\mathrm{cm}}^{-1}$. Figure 1c shows the complex permittivity of SiC. Between $\omega_{TO}$ and $\omega_{LO}$, the real part of the permittivity of SiC is negative, and light incident on SiC can form SPhP on the surface of SiC.

For the proposed structure, OTS is excited at the interface of the PhC heterostructure, and TPhP is excited at the interface of PhC2 and SiC, as shown in Figure 1d. The conditions for the two modes are, respectively [11]:

(2) $$r_{PhC1}r_{PhC2}\exp(2i\delta )=1,$$

(3) $$r_{PhC2}r_{SiC}=1,$$

where $r_{PhC1}$, $r_{PhC2}$, and $r_{SiC}$ represent the reflection coefficients of light at the interface of PhC1, PhC2, and SiC, respectively, which can be obtained by TMM. $\delta =2\pi n_{air}d_{0}/\lambda$ is the phase change of the light propagating across the air layer.

When infrared light is incident on the structure, a strong coupling phenomenon between OTS and TPhP will be observed. We use the coupled harmonic oscillator model to discuss the strong coupling phenomenon [26,27]:

(4) $$\left(\begin{array}{cc} E_{OTS}+i\hbar \Gamma_{OTS} & V\\ V & E_{TPhP}+i\hbar \Gamma_{TPhP} \end{array}\right)\left(\begin{array}{c} \alpha_{O}\\ \alpha_{T} \end{array}\right)=E\left(\begin{array}{c} \alpha_{O}\\ \alpha_{T} \end{array}\right),$$

where $\omega_{OTS}$, $E_{OTS}$, $\Gamma_{OTS}$ and $\omega_{TPhP}$, $E_{TPhP}$, $\Gamma_{TPhP}$ represent the angular frequency, energy and damping losses of OTS and TPhP modes, respectively, $E_{OTS}=\hbar \omega_{OTS}$ and $E_{TPhP}=\hbar \omega_{TPhP}$. E represents the energy of the coupled mode. ${\left|\alpha_{O}\right|}^{2}$ and ${\left|\alpha_{T}\right|}^{2}$ represent the relative weightings of OTS and TPhP modes in the coupled mode, ${\left|\alpha_{O}\right|}^{2}+{\left|\alpha_{T}\right|}^{2}=1$. *V* denotes interaction potential between two modes. When $\omega_{OTS}=\omega_{TPhP}$, the Rabi splitting energy is $\hbar \Omega =\sqrt{4V^{2}-\hbar^{2}{\left(\Gamma_{OTS}-\Gamma_{TPhP}\right)}^{2}}$.

The eigenfrequency of the coupled mode can be solved by Equation (4) [28,29]:

(5) $$\omega_{\pm }=\frac{\omega_{OTS}+\omega_{TPhP}}{2}\pm \sqrt{\omega_{0}^{2}+{\left(\frac{\omega_{OTS}-\omega_{TPhP}}{2}\right)}^{2}},$$

where $\omega_{\pm }$ represents the resonant angular frequencies of the coupled mode, $\omega_{OTS}$ and $\omega_{TPhP}$ are the resonant angular frequencies of OTS and TPhP modes, respectively, and $\omega_{0}=\Omega /2$ is the half of the Rabi splitting frequency.

## 3. Results and Analysis

### 3.1. Exciting of OTS and TPhP Modes

**Figure 2:**
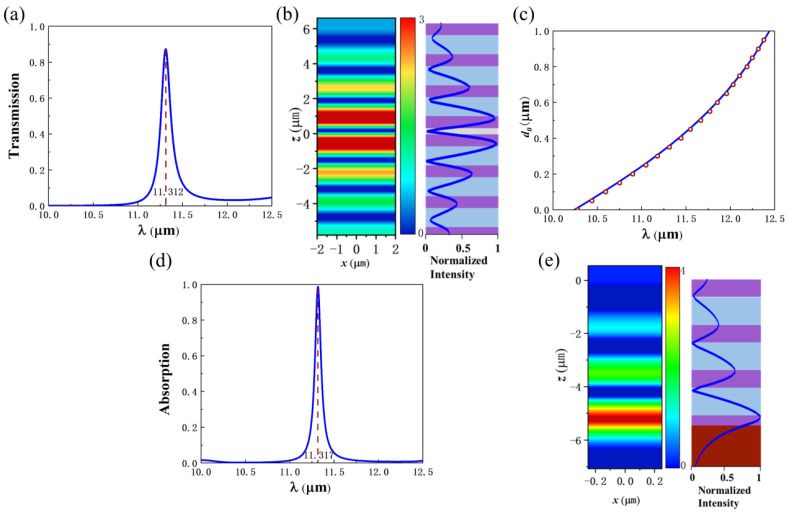
(**a**) The transmission spectrum of the OTS mode produced by the PhC heterostructure. (N_1_ = 3, N_2_ = 3, and d_0_ = 0.35 μm). (**b**) The electric field intensity distribution of OTS at 11.312 μm. (**c**) The relationship between the resonance wavelength of the OTS and the air layer thickness (N_1_ = 3, N_2_ = 3). (**d**) The absorption spectrum of the PhC2-SiC structure. (N_2_ = 3) (**e**) The electric field intensity distribution of TPhP at 11.317 μm (N_2_ = 3).

The structure consists of two parts. The upper part is composed of PhC1, an air layer, and PhC2, forming a PhC heterostructure. The lower part is composed of PhC2 and SiC. PhC2 acts as a common part. When the incident light illuminates the upper part of the structure, the OTS mode is excited at the heterogeneous interface, producing a sharp peak in the transmission spectrum. The resonance peak can be adjusted by changing the thickness of the air dielectric layer. Figure 2a shows the transmission spectrum of the PhC heterostructure when N_1_ = 3, N_2_ = 3, and d_0_ = 0.35 μm. The transmission peak is located at λ=11.312 μm. For λ=11.312 μm, $r_{PhC1}=-0.973+0.185i$ and $r_{PhC2}=-0.959+0.206i$ are obtained by TMM, and then $\left|r_{PhC1}\right|=0.99$, $\left|r_{PhC2}\right|=0.98$, $\varphi_{PhC1}=2.954$, and $\varphi_{PhC2}=-3.353$. $\left|r_{PhC1}\right|\left|r_{PhC2}\right|=0.97\approx 1$, which satisfies the amplitude condition of Equation (2). For λ=11.312 μm and d_0_ = 0.35 μm, $2\delta =2\times 2\pi n_{air}d_{0}/\lambda =0.388$, the total phase change is $\varphi =\varphi_{PhC1}+\varphi_{PhC2}+2\delta =-0.01\approx 0\pi$, which satisfies the phase condition of Equation (2). Figure 2b shows the electric field intensity distribution at 11.312 μm, when d_0_ = 0.35 μm. Most of the energy of the electric field is concentrated near the opposite PhC interface. The relationship between the wavelength of the transmission peak and d_0_ is shown in Figure 2c. The line represents the result calculated by Equation (2), and the circle represents the simulation result. When the air layer thickness d_0_ changes from 0 to 1.0 μm, the wavelength of the transmission peak will change from 10.24 μm to 12.44 μm. In fact, other dielectric materials can be used as separating layer, which will cause different resonant frequency of the OTS.

Figure 2d shows the absorption spectrum of the lower structure PhC2-SiC (where N_2_ = 3). The absorption peak is at a wavelength of λ=11.317 μm. For λ=11.317 μm, $r_{PhC2}=-0.748+0.63i$ and $r_{SiC}=-0.744-0.64i$ are obtained by TMM, and then $\left|r_{PhC2}\right|\left|r_{SiC}\right|=0.96\approx 1$, and $\varphi =\varphi_{PhC2}+\varphi_{SiC}=0.01\approx 0\pi$, which satisfy the amplitude and phase conditions of Equation (3). Figure 2e shows the electric field intensity distribution at 11.317 μm. The maximum electric field intensity appears near the interface between PhC2 and SiC, which is a TPhP mode.

### 3.2. Coupling of OTS and TPhP Modes

**Figure 3:**
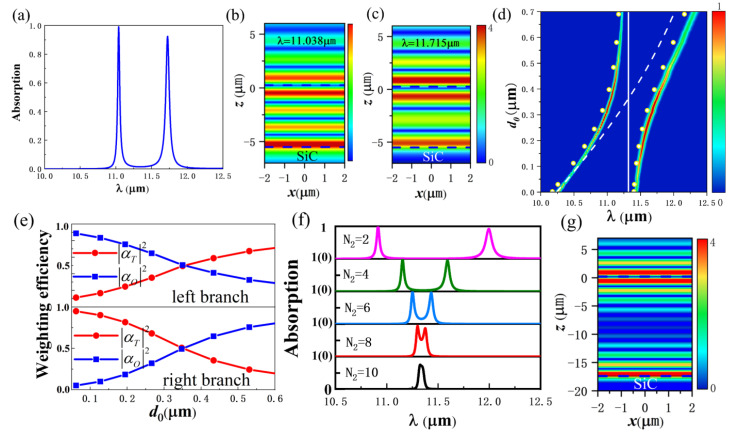
(**a**) The absorption spectrum of PhC1-air layer-PhC2-SiC structure. (**b**) The electric field intensity distribution at 11.038 μm. (**c**) The electric field intensity distribution at 11.715 μm. (**d**) The absorption spectra of OTS and TPhP coupled modes as a function of d_0_. (**e**) The mixing fractions of OTS and TPhP as a function of d_0_. (**f**) The absorption spectra of the structure when the period number N_2_ of PhC2 is different. (**g**) When N_2_ = 10, the electric field intensity distribution of the structure at the resonance wavelength.

Figure 3a shows the absorption spectrum of the structure of Figure 1a,b (N_1_ = 3, N_2_ = 3, d_0_ = 0.35 μm). Due to the strong coupling of OTS and TPhP, the original overlapping peaks split into two absorption peaks, located at λ=11.038 μm and λ=11.715 μm, respectively. Figure 3b,c are the electric field intensity distributions at 11.038 μm and 11.715 μm, respectively. The dashed lines in the figure indicate the middle of the air layer in the PhC heterostructure and the interface of PhC2-SiC. The electric field energy is mainly distributed in the air gap at the interface of heterostructure and near the interface of PhC2 and SiC. There are similar electric field distributions at the two wavelengths, corresponding to the coupling mode of OTS and TPhP.

By adjusting the thickness d_0_ of the air layer, the detuning of OTS and TPhP can be adjusted to affect the position of the absorption peak and the absorption intensity. It can be seen from Figure 2c that, when d_0_ increases from 0 to 1.0 μm, the resonance wavelength of OTS changes from 10.24 μm to 12.44 μm. Figure 3d shows the absorption spectra of the coupling mode of OTS and TPhP when d_0_ changes. The dashed line and solid line, respectively, represent the resonant wavelengths of the OTS mode and the TPhP mode when two modes are not coupled. They are calculated by using Equations (2) and (3). Obviously, when the d_0_ changes, the resonance wavelength of TPhP will not change.

The absorption spectra of the coupled mode show an anti-crossing property near 11.315 μm (d_0_ = 0.35 μm), and the energy of Rabi splitting is 6.5 meV. Figure 3e shows the weight of OTS and TPhP of the coupled mode calculated according to Equations (4) and (5). When d_0_ = 0.35 μm, ${\left|\alpha_{O}\right|}^{2}={\left|\alpha_{T}\right|}^{2}=0.5$. As d_0_ decreases, the left branch appears to have an OTS-like characteristic (${\left|\alpha_{O}\right|}^{2}>{\left|\alpha_{T}\right|}^{2}$). With the increase of d_0_, the left branch appears to have a TPhP-like characteristic (${\left|\alpha_{O}\right|}^{2}<{\left|\alpha_{T}\right|}^{2}$). Similarly, with the decrease of d_0_, the right branch appears to have a TPhP-like characteristic, and, as d_0_ increases, the OTS-like characteristic appears. The eigenfrequencies of the coupled mode can also be calculated theoretically using the coupled harmonic oscillator model by Equations (4) and (5), and they are represented by yellow dots in Figure 3d.

Next, we study the effect of PhC2 period number N_2_ on coupling when N_1_ = 3 and d_0_ = 0.35 μm. It can be seen from Equations (2) and (3) that, when only N_2_ is changed, the resonance conditions of the two modes will remain unchanged, and the zero detuning state can still be maintained. Figure 3f shows the absorption spectra when the number of period of PhC2 increases from N_2_ = 2 to N_2_ = 10. For smaller N_2_, the OTS field energy can easily pass through PhC2 to reach the SiC interface, and TPhP can interact strongly with OTS, leading to large Rabi splitting. The absorption spectrum can be well fitted to two Lorentz resonance lines. With the increase of the PhC2 period, the energy of OTS reaching the SiC interface decreases, and the coupling intensity between OTS and TPhP decreases gradually. When N_2_ = 10, the distance between the heterostructure and the SiC is far enough that OTS and TPhP cannot exchange energy (namely, there is no coupling), and the absorption spectrum shows a Lorentz single resonance line. In this case, the electric field distribution of the structure is shown in Figure 3g. Both OTS and TPhP modes are excited, but there is no energy exchange between them.

### 3.3. Multi-Channel Adjustable Absorber

**Figure 4:**
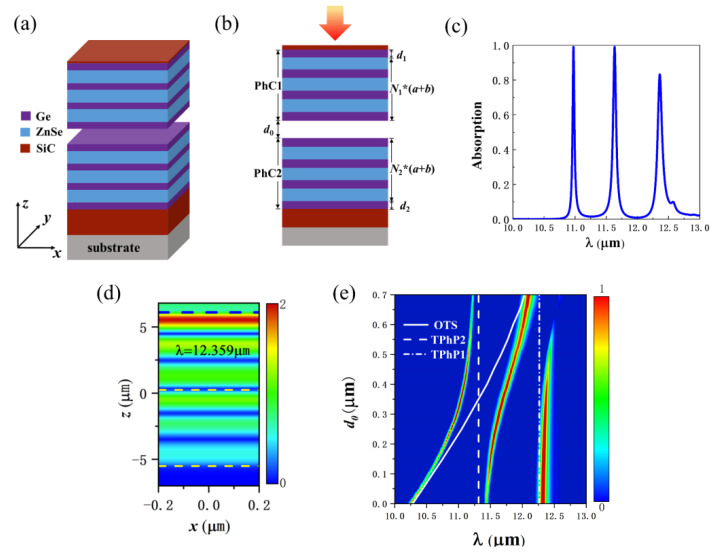
(**a**) Schematic diagram of the structure of SiC-PhC heterostructure-SiC structure. *N*_1_*(*a+b*) and *N*_2_*(*a+b*) indicate that the number of periodic layers of PhC is *N*_1_ and *N*_2_, respectively. (**b**) Side view of the structure. (**c**) The absorption spectrum of SiC-PhC heterostructure-SiC structure (d_0_ = 0.3 μm). (**d**) The electric field intensity distribution at 12.359 μm. (**e**) The absorption spectra of SiC-PhC heterostructure-SiC structure as a function of d_0_.

Based on the basic structure, we add a 0.2 μm SiC layer to the upper layer of PhC1, as shown in Figure 4a,b. When d_0_ = 0.3 μm, the absorption spectrum of the structure is shown as in Figure 4c. There are three absorption peaks, at $\lambda_{1}=10.974$ μm, $\lambda_{2}=11.634$ μm, and $\lambda_{3}=12.359$ μm. They come from the coupling of three modes, namely OTS in the PhC heterostructure, TPhP2 at the PhC2-SiC interface, and TPhP1 at the SiC-PhC1 interface. At $\lambda_{1}=10.974$ μm and $\lambda_{2}=11.634$ μm, the electric field distributions are similar to those in Figure 3b,c, although not given in the article. Figure 4d shows the electric field distribution at 12.359 μm. The dashed lines from top to bottom represent the interface between SiC and PhC1, the middle of the air layer, and the interface between PhC2 and SiC. The electric field energy is mainly distributed near the interface between SiC and PhC1, so $\lambda_{3}$ mainly comes from the TPhP1 mode formed by SiC and PhC1. Figure 4e shows the absorption spectra of the structure varying with the thickness of the air layer. As shown in Figure 2c, when there is no coupling, the OTS redshifts with increasing d_0_. When coupling occurs, due to the anti-crossing characteristics, the three branches of the coupled mode redshift with the increase of d_0_. When d_0_ increases to about 0.6 μm, the rightmost branch is beyond the reststrahlen band of SiC, 10.28~12.55 μm. In this case, the real part of the permittivity of SiC changes from negative to positive. The incident light cannot excite TPhP1 mode, so the $\lambda_{3}$ absorption peak disappears. The number and position of absorption peaks can be adjusted by adjusting d_0_.

### 3.4. Influence of Incident Angle on Absorption Spectrum

**Figure 5:**
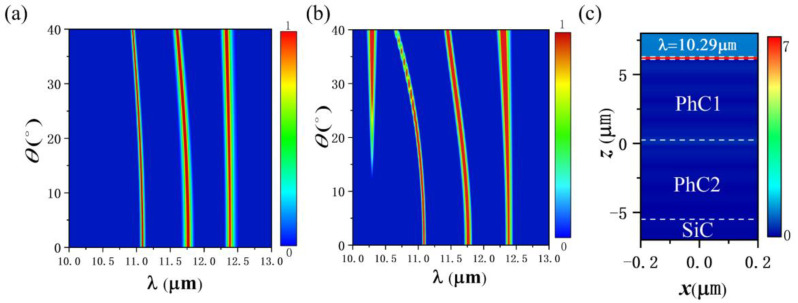
(**a**) The relationship between the absorption spectra and the incident angle for TE polarized light (N_1_ = 3, N_2_ = 3, d_0_ = 0.4 μm). (**b**) The relationship between the absorption spectra and the incident angle for TM polarized light (N_1_ = 3, N_2_ = 3, d_0_ = 0.4 μm). (**c**) The electric field intensity distribution at 10.29 μm.

When light is incident perpendicularly to the structure, the resonance modes formed by TE and TM polarization lights degenerate. When light is incident on the structure at a certain angle of incidence, the degeneracy is eliminated. Both OTS and TPhP modes are related to the incident angle, so changing the incident angle can adjust the position and intensity of the absorption peak. Figure 5a,b show the relationship between the absorption spectra and the incident angle when TE and TM polarized lights are incident (N_1_ = 3, N_2_ = 3, d_0_ = 0.4 μm). As the incident angle increases, the position of each absorption peak is blue-shifted. This is because, when the light changes from normal incidence to oblique incidence, the phase changes through each layer from φ=2πnd/λ to φ=2πndcosθ/λ (θ is the angle of incidence). In order to satisfy the phase condition of Equations (2) and (3), the resonance wavelength is blue-shifted. The larger the θ, the more obvious the blue shift is. For TM polarized light, as incident angle increases, a new absorption peak appears at λ=10.29 μm, and the absorption rate increases as the incident angle increases.

When TE polarized light is incident on the ultra-thin SiC film, the electric field only has a *y*-direction component, which can only excite the transverse SPhP. When TM polarized light is normally incident on the ultra-thin SiC film, the electric field only has an *x*-direction component, and only the transverse SPhP is excited. When TM polarized light is obliquely incident on the ultra-thin SiC film, the electric field has components in *z* and *x* directions, and the transverse SPhP and the longitudinal SPhP may be excited. The longitudinal SPhP frequency is close to the longitudinal optical phonon frequency (10.28 μm) [30,31,32]. At this frequency, the real part of the dielectric constant of SiC is close to zero, forming ENZ polariton [15,33]. It has a strong sub-wavelength limitation, which greatly enhances the electric field intensity in the film and forms efficient absorption. The absorption peak at λ=10.29 μm is ENZ mode, its electric field distribution is shown in Figure 5c, in which the dashed lines are the separators of each part, and the top two dashed lines represent the upper and lower sides of the SiC layer. The electric field energy is almost all distributed in the SiC layer. Moreover, the intensity of the longitudinal SPhP is proportional to $\sin^{2}\theta$, θ is the angle of incidence. The absorption spectrum can be adjusted by adjusting the polarization state and incident angle of the incident light.

## 4. Conclusions

A SiC-PhC heterostructure-SiC structure is proposed to realize the tunable multi-channel absorption in the mid-infrared. The absorption characteristics of the structure are studied from two aspects of numerical simulation and theoretical calculation. The effects of structural parameters, polarization state of incident light and incident angle on the absorption spectrum are analyzed in detail. The results show that the structure can achieve multiple absorption peaks due to the coupling effect between OTS and TPhP excited at the interface. Adjusting the thickness of the air layer at the junction of the PhC heterostructure can regulate the resonance wavelength of the OTS mode, thereby controlling the detuning between the OTS and TPhP modes, and controlling the position and intensity of the absorption peaks. Changing the period of PhC2 in the PhC heterostructure can adjust the coupling strength of OTS and TPhP modes, as well as adjust the position and number of absorption peaks. When the incident light is obliquely incident on the structure, the degeneracy of TM and TE polarized light is eliminated. In the case of TM polarization light oblique incidence, ENZ mode will also be generated, forming an efficient absorption peak. This design will have potential applications in filters, heat radiators, sensors, etc.

## Data Availability

The study did not report any data.
